# Development of Rectal Ulcer Leading to Hematochezia After Placement of SpaceOAR Hydrogel for Prostate Carcinoma: A Case Report and Review of Literature

**DOI:** 10.7759/cureus.95926

**Published:** 2025-11-01

**Authors:** Janak Bahirwani, Purna Chand Paidi, Shrivatsam Paidi, Brittney Shupp, Ronak Modi

**Affiliations:** 1 Gastroenterology, Kadlec Regional Medical Center, Richland, USA; 2 Medicine, Washington State University, Richland, USA; 3 Internal Medicine, Andhra Medical College, Visakhapatnam, IND; 4 Medicine, Government Medical College, Srikakulam, Srikakulam, IND; 5 Gastroenterology, St. Luke's University Health Network, Bethlehem, USA

**Keywords:** hematochezia, prostate cancer, radiotherapy, rectal ulcer, spaceoar hydrogel

## Abstract

SpaceOAR hydrogel is a synthetic polyethylene glycol-based spacer used to reduce rectal radiation exposure in patients undergoing radiotherapy (RT) for prostate cancer. While generally safe, rare complications such as rectal ulceration have been reported. We describe a 76-year-old male patient with prostate adenocarcinoma who underwent SpaceOAR hydrogel and fiducial marker placement prior to RT. Within 10 days of the procedure, he developed severe rectal pain and hematochezia. Computerized tomography (CT) imaging showed focal proctitis adjacent to the spacer, and colonoscopy revealed a large anterior rectal ulcer with fibrotic tissue at the site of spacer placement. He was managed conservatively with antibiotics and laxatives, with complete symptom resolution. Early-onset rectal ulceration after hydrogel insertion is extremely rare, with only a few cases reported; most cases in the literature occur weeks to months later. The mechanism is likely multifactorial. In contrast to prior case reports, our patient presented within 10 days, making this one of the earliest documented cases. This highlights the need to maintain a high index of suspicion for such complications to ensure timely diagnosis and treatment, enabling prompt resumption of RT.

## Introduction

SpaceOAR hydrogel is a soft, gel-like synthetic material composed of polyethylene glycol (PEG), which was FDA-approved in 2015 for prostate cancer patients who are planning to undergo radiotherapy (RT). It is intended to position the anterior rectal wall away from the prostate during RT, thereby reducing the dose of radiation delivered to the rectum and minimizing complications. The hydrogel is typically injected transperineally under transrectal ultrasound guidance and remains in place for approximately three to six months, after which it is naturally absorbed by the body [1].

RT is a standard treatment for prostate cancer, which is frequently encountered worldwide. During RT, high doses of radiation are administered to the rectum and bladder, which surround the prostate; this can result in side effects such as radiation proctitis and radiation cystitis. Placing a SpaceOAR hydrogel spacer allows for greater separation between the prostate and rectum, enabling higher doses of radiation to be delivered to the prostate while reducing collateral damage to surrounding tissues [2]. We present a rare case of a rectal ulcer that developed after implantation of a SpaceOAR hydrogel spacer prior to RT. Such cases may become more common as the use of hydrogel spacers increases alongside the rising incidence of prostate cancer. Additionally, we reviewed the existing literature on rectal ulcers associated with post-RT RT even with hydrogel spacer insertion. This article was previously presented as a meeting abstract at the 2022 American College of Gastroenterology (ACG) conference.

## Case presentation

A 76-year-old male patient with chronic kidney disease, recently diagnosed prostate adenocarcinoma (Gleason score 3+4) was scheduled to undergo RT. To reduce rectal toxicity after RT, he underwent transperineal implantation of a SpaceOAR hydrogel spacer and gold fiducial markers prior to RT. Approximately 10 days after the procedure, he developed severe rectal pain accompanied by hematochezia. On physical examination, his vital signs were stable, and his abdomen was soft and nontender with no signs of peritonitis. A digital rectal examination revealed the presence of bright red blood per rectum. Laboratory investigations showed a normal hemoglobin level of 16 g/dL and other laboratory parameters including a metabolic panel were within normal limits.

**Figure 1:**
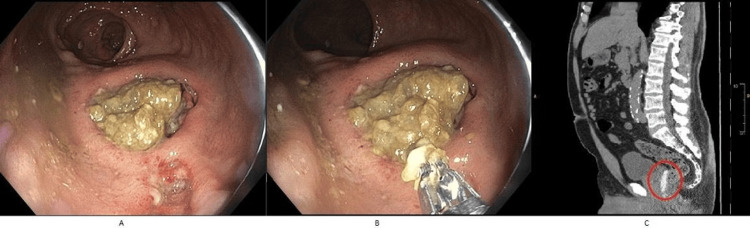
Imaging and endoscopic features of rectal ulceration following SpaceOAR hydrogel insertion (A) Colonoscopy image showing focal proctitis and a large ulcer on the anterior rectal wall. (B) Friable rectal mucosa at the ulcer site, not amenable to biopsy. (C) Axial CT scan showing hydrogel spacer between the prostate and anterior rectal wall with signs of focal proctitis

A noncontrast computed tomography (CT) scan of the pelvis demonstrated appropriate placement of the hydrogel spacer between the posterior prostate and the anterior rectal wall. However, focal thickening of the anterior rectal wall consistent with proctitis was also observed (Figure 1C). Due to persistent hematochezia, a colonoscopy was performed. It revealed focal proctitis, as noted on imaging, and also identified a large rectal ulcer with overlying fibrotic tissue on the anterior rectal wall, corresponding to the site of recent instrumentation (Figure 1A). The tissue appeared friable and was not amenable to biopsy (Figure 1B).

The patient was managed conservatively with a 10-day course of oral levofloxacin and polyethylene glycol to maintain soft bowel movements and prevent straining. Over subsequent follow-up visits, he reported resolution of rectal bleeding and significant improvement in rectal pain. After complete symptom resolution, plans were made to initiate definitive RT for prostate cancer. A repeat flexible sigmoidoscopy performed six weeks after the initial presentation showed that the ulcer had healed. 

## Discussion

RT remains a standard modality for the treatment of prostate cancer. However, radiation exposure to adjacent structures such as the rectum can result in adverse effects including fecal urgency, rectal bleeding, ulceration, incontinence, and, in rare cases, fistula formation. To mitigate this, the use of peri-rectal hydrogel spacers such as SpaceOAR has been adopted to displace the rectal wall from the high-dose radiation field [2]. Radiation proctitis is a challenging complication of RT, but it can be effectively prevented with the use of a hydrogel spacer [1]. The SpaceOAR is implanted by transperineal injection under ultrasound guidance. The hydrogel maintains this separation for approximately three months before being absorbed. According to the Common Terminology Criteria for Adverse Events version 4, the incidence of grade 2 or higher adverse events related to SpaceOAR occur in approximately 3.3%-4.1% of patients [1,3]. Only a few isolated cases of rectal ulceration attributable to hydrogel insertion have been reported, including the present case [4-9].

The current case adds to this literature by describing a patient who developed rectal ulceration and hematochezia only 10 days after hydrogel insertion, making it one of the earliest reported symptomatic presentations. In reviewing the literature, we identified six additional case reports describing rectal ulcers or related mucosal injury after SpaceOAR hydrogel insertion. Table 1 highlights all these case reports. These cases occurred in male patients aged 63-79 years, with various clinical stages and Gleason scores. Most patients presented with symptoms 1-3 months after insertion of hydrogel. Our patient developed symptoms of rectal pain and hematochezia just 10 days after hydrogel placement, which is notably earlier than the onset reported in previously published cases, which typically ranged from several weeks to months. CT and colonoscopy confirmed the presence of proctitis and a large anterior rectal ulcer. He was treated conservatively with antibiotics and laxatives, resulting in complete symptom resolution. RT was later initiated as planned. Among the six full-text accessible case reports, three patients were managed as outpatients, while three required inpatient care due to more severe presentations such as ischemic proctitis or rectal perforation. All patients eventually recovered, and RT was either resumed or continued after appropriate mucosal healing [4-9].

**Table 1:** Review of cases documented in literature (including ours) presenting with complications due to hydrogel placement RT: radiotherapy; CT: computed tomography; MRI: magnetic resonance imaging; EBRT: external beam radiotherapy; HDR: high dose rate; MRgRT: magnetic resonance-guided radiotherapy; M: months; W: weeks; Y: yes; N: no; HBOT: hyperbaric oxygen therapy

Case	Reporter	Year	Age (years)	Sex	Gleason score	Radiotherapy	Time from insertion to diagnosis	Time from diagnosis to mucosal improvement	History of sigmoid diverticula (Y/N)	Radiology	Colonoscopy	Management	Time from the insertion to resumption of radiotherapy
1	Teh et al. [4]	2014	66	M	3+4	Brachytherapy	2 M	1 M	Y	CT-not listed; MRI-not inspected	Ulcer at anterior rectal wall, no proctitis	Outpatient conservative management; no recurrence	Already treated
2	Iinuma et al. [5]	2019	63	M	4+3	Brachytherapy, external radiation therapy	1 M	1 M	Y	CT-air in space between prostate and rectum; MRI-rectal wall penetration	Ulcer at anterior rectal wall, no proctitis	Outpatient conservative management; no recurrence	3 M later
3	Imai et al. [6]	2020	75	M	3+3	External radiation therapy	6 M	34 days	N	CT-air between prostate and rectum; MRI-rectal wall infiltration	Ulcer with exposed vessel, no proctitis	Inpatient for 5 days due to ulcer with exposed vessel; conservative treatment; no recurrence	Already treated
4	Kashihara et al. [7]	2020	78	M	Unknown	EBRT followed by HDR	2 M	Healed by end of HBOT	Unknown	CT-abscess and perforation; MRI-not listed	Perforation site and ulcer	Inpatient due to rectal perforation and abscess; treated with HBOT; no recurrence	Resumed with MRgRT
5	Toriumi et al. [8]	2022	79	M	Unknown	Brachytherapy	2 M	19 days	Unknown	CT-not listed; MRI-not listed	Ischemic proctitis	Inpatient for 19 days due to ischemic proctitis; conservative management; no recurrence	Resumed after recovery
6	Yagi et al. [9]	2024	75	M	4+4	External radiation therapy	3 W	2 M	Y	CT-low density in perirectal space; MRI-high signal within rectal wall	Ulcer at anterior rectal wall, no proctitis	Outpatient conservative management; no recurrence.	4 M later
7	Present case	2025	76	M	3+4	Radiotherapy	10 days	Few weeks	Y	CT-hydrogel between prostate and rectum, focal proctitis; MRI-not done	Ulcer and proctitis	Outpatient conservative management with antibiotics and laxatives; no recurrence.	Scheduled after healing

The most consistent colonoscopic finding across reports was a solitary anterior rectal ulcer, often with fibrotic or friable tissue, located at the hydrogel implantation site as in our patient. CT and MRI findings varied, but many showed rectal wall thickening, proctitis, or direct hydrogel infiltration into the rectal wall. A history of sigmoid diverticula was noted in several cases and may represent a predisposing factor for mucosal injury, possibly due to local ischemia or altered rectal wall integrity. In our patient, no such history was present [4,5,9].

The pathophysiology underlying rectal ulcer formation after hydrogel spacer insertion is multifactorial and remains incompletely understood. Proposed mechanisms include mechanical trauma from the injection needle, ischemic injury due to excessive tension or pressure on the rectal wall, and, in rare cases, infection. Importantly, most reports, including ours, did not demonstrate evidence of radiation-induced proctitis. Teh et al. described a case where antimicrobial therapy was used empirically, but the role of infection remains speculative [4]. Similarly, our case involved prophylactic antibiotic administration, but no definitive signs of infection were identified.

The etiology behind such complications may also involve subtle technical factors during the procedure. For instance, the gel might have been placed too close to the rectal wall, or variations in injection technique could have led to unexpected trauma or pressure [4,5]. There's even a theoretical possibility that the gel alters how radiation is absorbed or distributed, potentially affecting nearby tissue [9]. In some cases, the spacer may not achieve adequate separation between the rectum and prostate, reducing its intended protective effect [6]. Since these mechanisms are not fully understood and may be multifactorial, it’s important to remain vigilant. When patients' present with symptoms like rectal pain or hematochezia soon after hydrogel insertion, clinicians should have a low threshold to evaluate for such complications using imaging or colonoscopy, ensuring that mucosal healing is underway before initiating or resuming RT [8,9].

In some instances, direct rectal wall infiltration or needle misplacement has been documented via MRI or colonoscopy [10]. Additionally, patients with preexisting sigmoid diverticula may be more susceptible to local tissue vulnerability. In our compiled cases, three had documented sigmoid diverticulosis. Diverticular disease may contribute to localized inflammation, fibrosis, or adhesions, potentially exacerbating ischemic injury after hydrogel expansion. However, the sample size remains limited, and further studies are required to confirm this association.

Importantly, RT was delayed in some cases until mucosal healing was confirmed, while in others it proceeded without interruption [4,5,8]. In our patient, hematochezia resolved and pain improved following antibiotic and laxative treatment, and RT was able to proceed as scheduled. Early recognition and intervention are therefore critical to avoiding prolonged treatment delays and ensuring cancer control [5].

## Conclusions

As the use of SpaceOAR increases in clinical practice and prostate cancer incidence rises, more such complications may be encountered. Therefore, early recognition and appropriate imaging are critical. MRI or CT should be considered within the first 1-3 months following spacer insertion, especially in patients with predisposing factors such as diverticulosis or unexplained rectal symptoms. Additionally, clinicians should maintain a high index of suspicion for ulceration in patients presenting with rectal pain or hematochezia after spacer placement, even in the absence of prior RT. In conclusion, we present a rare but clinically significant complication of rectal ulceration shortly after SpaceOAR hydrogel spacer placement in a patient with prostate cancer. While hydrogel spacers are effective in reducing radiation proctitis, they may themselves cause rectal injury via mechanical or ischemic mechanisms. Early endoscopic and radiologic evaluation, along with conservative management, can lead to favorable outcomes and allow for timely resumption of cancer-directed therapy.
